# Dilemma during ultrasound‐guided internal jugular venous catheterization

**DOI:** 10.1002/ccr3.1117

**Published:** 2017-09-05

**Authors:** Sanjay Dwarakanath, Monica Cheriyan, Annette Rebel

**Affiliations:** ^1^ Department of Anesthesiology University of Kentucky Lexington Kentucky; ^2^ Present address: Cleveland Clinic Regional Practice Anesthesiology 9500 Euclid Avenue Cleveland 44195 Ohio

**Keywords:** Internal jugular vein, ultrasound, venous cannulation, venous valves

## Abstract

The presence of Internal Jugular Valves can pose a diagnostic and procedural challenge during ultrasound‐guided cannulation. After ruling out dissection, thrombus, or ultrasound artifacts, it can still be accessed and successfully cannulated with appropriate precautions including use of Live ultrasound, positioning, use of soft‐tipped catheters, and minimizing duration of catheter placement.

## Case

**Figure 1:**
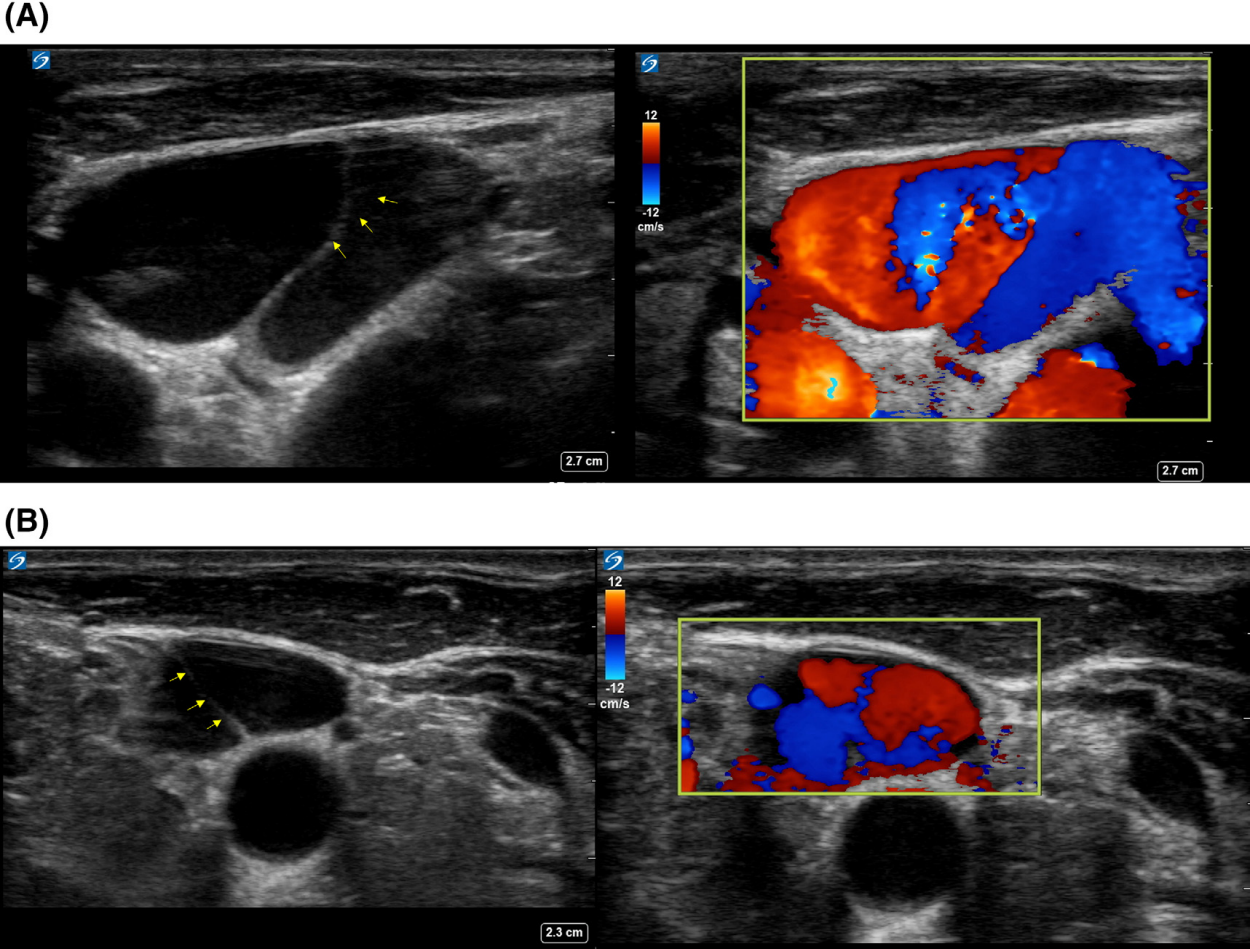
Ultrasound image of (A) right (B) left Internal Jugular Vein. 2D image seen on the left display and Color Doppler evaluation seen on the right display. Arrows point to the mobile structure seen within the lumen of the vein.

A 67‐year‐old man admitted to the Intensive Care Unit for management of pneumonia with Septic Shock, on mechanical ventilator support was undergoing a central line cannulation in his right internal jugular vein (IJV) using ultrasound guidance. After skin sterilization and draping, a freely mobile structure in right IJV (Fig. 1A, Video S1) was visualized at a level 1 cm proximal to the clavicle. A similar looking image was seen on the left IJV as well (Fig. 1B).

## Question

What is your diagnosis and what would you do next?

## Discussion

The presence of similar looking mobile flap on both sides of the internal jugular vein (IJV) is suggestive of IJV valves 1. Although dissection is possible, it is unlikely in the absence of previous intervention. Collapsibility, the presence of flow in entire lumen (using Color Doppler) and the absence of echogenic mass filling the lumen can help rule out presence of thrombus. IJV can still be accessed for catheterization in the presence of a valve, although the risks including thrombus and valve incompetency should be kept in mind. Risk versus benefit should be weighed before selecting other sites for central venous cannulation. To avoid direct injury to the valve, IJV should be accessed during live ultrasound visualization, with venipuncture performed at a level distal to the extension of the valve (usually cricoid ring or higher in the neck) using a soft‐tipped flexible guidewire 2. In an upright position, the valves are more open and saddled to the wall, hence a reverse Trendelenburg position may further facilitate catheter placement 3.

In our case, the right IJV was successfully cannulated with a 7 Fr catheter at the level of cricoid cartilage. Cannula was removed 3 days later. A follow‐up ultrasound evaluation 3 months after demonstrated unchanged anatomy without any evidence of thrombus formation.

## Authorship

SD: involved in manuscript preparation, analysis of data, and acquisition of images. MC: involved in analysis of data and acquisition of images. AR: involved in manuscript preparation, analysis of data, and acquisition of images.

## Conflict of Interest

None declared.

## Supporting information


**Video S1.** Live 2D image of the right IJV. IJ valve is seen as a mobile, echogenic structure traversing within the lumen of the vein.
